# The legacy of traditional rice cultivation by descendants of Indian contract laborers in Suriname

**DOI:** 10.1186/s13002-021-00485-6

**Published:** 2021-10-18

**Authors:** Melissa Ramdayal, Harro Maat, Tinde van Andel

**Affiliations:** 1 Institute of Biology, Leiden University, Leiden, The Netherlands; 2 Social Science Department, Wageningen University, Wageningen, The Netherlands; 3 Naturalis Biodiversity Center, Leiden, The Netherlands; 4 Biosystematics Group, Wageningen University, Wageningen, the Netherlands

**Keywords:** Indentured labor, Indian diaspora, Landraces, Maroons, Oral history, *Oryza sativa*

## Abstract

**Background:**

Some 35,000 indentured laborers from India were recruited to work on plantations in Suriname between 1868 and 1916. It is likely that most were familiar with farming before they were shipped to this former Dutch colony in the Caribbean. Around 1900, those who did not return received a piece of land where most of them started growing rice as a staple crop. Agronomists characterized their traditional landraces as inferior and infested with weedy rice and started to ‘purify’ these landraces. No research has been done on whether these ancient rice varieties still exist. We aimed to document the rice varieties (both landraces and more modern cultivars) grown currently or in the recent past by (descendants of) Hindustani smallholders in Suriname, their origin, morphological and agronomic characters, local uses and cultural and spiritual relevance. Given the rapid decline in small-scale rice cultivation in the past 40 years, we wanted to know why people continued or abandoned rice farming and what aspects of traditional practices still survived.

**Methods:**

We interviewed 26 (former) small-scale Hindustani farmers and asked about the varieties they cultivated and traditional agricultural practices. We collected seed samples, local names and associated information, and compared these to information from agricultural reports from the colonial period. We also interviewed 11 Maroons, one Javanese farmer, and three persons of mixed ethnicity, who were somehow involved in the cultivation of East Indian rice varieties.

**Results and discussion:**

Hindustani smallholders in Suriname largely lost their traditional rice landraces. Most of the interviewed farmers grew modern cultivars, developed after 2000. Some cultivars from the 1950s were still planted for fodder, but these were heavily mixed with weedy rice and other weeds. Maroon farmers in the interior, however, still actively cultivated varieties with names like ‘coolie rice’, which probably descend from landraces introduced by the Indian contract laborers, although this needs to be confirmed by molecular research. Although traditional cultivation practices seem to have been lost, smallholders still retain pleasant memories of the manual planting, harvesting, and processing of rice, as well as the gender-based practices and beliefs associated with the cultivation of the crop. The oral history of former rice farmers and traditional rice varieties (possibly obtained from Maroon fields) could play a role in museum settings as living vehicles for memories of the descendants of Asian contract labourers in Suriname and Guyana.

**Supplementary Information:**

The online version contains supplementary material available at 10.1186/s13002-021-00485-6.

## Background

From 1838 to 1917, over half a million citizens from the former British India were recruited as indentured workers on Caribbean sugarcane plantations to address the labor shortage following the abolition of slavery. These movements of people, their impact on the plantation economy and the social and cultural dynamics in the Caribbean are well documented [1, 2]. However, less is known about the production of food for their own subsistence, although colonial agricultural reports mention the many traditional crops that the Asian contract laborers brought from their home countries and cultivated in their small fields and house yards [3, 4].

In Suriname, a former Dutch colony in northern South America that can be characterized culturally as Caribbean, the first ‘British Indians’ entered via Guyana in 1868, just five years after slavery was abolished in 1863. A ship named *Lalla Rookh* arrived in Paramaribo on 5 June 1873, carrying 339 immigrants who had boarded in Calcutta, coming from the current regions of Uttar Pradesh, Uttarakhand and Bihar in northern India. In 1916 migration from India came to an end after the arrival of c. 35,000 East Indians in Suriname, who refer to themselves today as ‘Hindustanis’ [2], but were indicated in the past as ‘koelies’ (coolies) or kantráki, the Sranantongo word for contract laborers [1]. Between 1890 and 1940, the Dutch also recruited 32,956 Javanese to work in Suriname [2, 5]. Most Asian laborers were recruited on the basis of a three-year contract. Working conditions on the plantations were harsh and payment was low, so when their contract ended, few enlisted for a second contract, and about one third returned to their home countries. The majority, however, stayed in Suriname, and continued as smallholder farmers, initially squatting on land but later stimulated by the colonial government through a land allocation regulation [6, 7]. From about 1910, the area farmed by smallholders was larger than that of the plantation sector [5]. Currently, the Hindostani make up c. 23% of the population of Suriname [1].

Dutch colonial agronomists observed that ‘most of the British Indians were growing rice as a staple crop’ [8]. In spite of the ‘very primitive and extremely flawed manner in which these people cultivate these crops’ [6:342], they envisioned that the rice produced (all Asian rice, *Oryza sativa* L.) could meet Suriname’s domestic demand and even become an export crop. Plans were made for mechanized rice farming and replacement of the traditional, ‘non-commercial’ rice landraces by improved cultivars suitable for machine-harvesting [6, 8].

In neighboring Guyana, initiatives towards commercial rice farming started much earlier: in 1895, the first rice mill was established in Georgetown, but failed as the rice supplied by East Indian farmers consisted of many different varieties and grain sizes [9]. Around 1908, a series of smaller mills were operating in the coastal villages and agricultural instructors were sent out to teach farmers to become more commercially oriented. By advancing them cash and goods, the rice mill owners drew the farmers into debt and forced them to abandon their traditional, self-developed landraces, as they paid higher prices for uniform ‘pure’ seed [9]. In 1927, traditional East Indian rice varieties were tested for their suitability for large-scale mechanical cultivation in the Experimental Station in Georgetown. Although landraces were generally characterized as inferior, infested with weedy red rice and mixed with odd-looking types, some were selected as promising material to breed new cultivars in 1933 [10].

From 1904 onwards, agronomists in Suriname imported about 50 rice varieties from Demerara (Guyana), India, Java and French Indochina (Vietnam) and tested them in the coastal districts [11–13]. Only few of the imported varieties performed well on the heavy clay soils [13]. Smallholders from Indian origin had different preferences than those from Java [12]. Popular Hindustani rice types were Moetmoeria, Fini tere, Djerehi, and Skrivimankoti, the latter was considered to be the most interesting commercial variety [13].

After World War II, large areas of the coastal wetlands of Nickerie were transformed into commercial rice fields, operated by the state-sponsored SML (Stichting Machinale Landbouw) and the Van Dijk company. The scheme was run on the basis of contracts with mostly Hindustani farmers [3]. After 1965, the semi-dwarf varieties from the International Rice Research Institute (IRRI) were included in the breeding program. After independence in 1975, the Surinamese National Rice Institute (SNRI/ADRON) continued to breed modern high-yielding cultivars for the rice sector [7, 14]. In 2014, over 35,000 hectares were cultivated by farms of 12 hectares or more, whereas smaller farms totaled to about 26,000 hectares, together producing 275,000 tons of which c. 105,000 tons were exported (http://suriname-rice.com/rice-statistics/). Almost every rice farmer in coastal Suriname produces for national and international markets, but there is quite some variation in farm size, cropping patterns and uses of rice. Hardly any research has been done on rice varieties cultivated by smallholder farmers after the 1960s [15].

Landraces are farmer-developed, traditional crop varieties that are genetically variable and thus adaptable to local climate and soil conditions and cultural preferences [16, 17]. Thousands of rice landraces were grown before the advent of the Green Revolution, but the modernization of agriculture resulted in a dominance of a limited number of cultivars, developed by breeding institutes, that yield well due to chemical fertilizers, pesticides and controlled water supply. Since the 1950s, a significant proportion of rice landraces has disappeared from farmer’s fields worldwide [18]. Traditional landraces are increasingly considered as an untapped genetic resource for breeding new cultivars resilient to future challenges [16, 19]. Apart from the genetic and agronomic potential, landraces also contribute to the understanding of cultural heritage, including human migration trade and crop exchanges between communities [20–22].

An example from Suriname is a black-seeded type of *Oryza sativa*, known as ‘Ketan iran’ observed in 2012 in a wetland field near the former plantation Reijnsdorp (Commewijne district). The farmer, an aged Javanese lady, said she was the only one who still planted this once popular type of dark glutinous rice, which she used for food and ceremonies [23]. Sticky Javanese rice types entered Suriname between 1907 and 1911 [13], but were gradually replaced by other varieties in 1929. Another example is the encounter of African rice (*O. glaberrima* Steud.) in a rice field of Maroons, descendants of escaped slaves living in the forested interior, originating from western Ivory Coast [22, 24].

The aim of this research was to document the rice varieties grown currently or in the past decades by (descendants of) Hindustani smallholders in coastal Suriname, their origin, morphological and agronomic characters, local uses and cultural and spiritual relevance. Given the rapid decline in small-scale rice cultivation in coastal Suriname in the past 40 years [25], we wanted to know why people continued or abandoned rice farming and what aspects of traditional practices still survived. We focused specifically on indications of rice exchange between Hindustani farmers and other ethnic groups in Suriname, such as Javanese and Maroons. We hope that this study contributes to the preservation of oral and agricultural history among descendants of East Indian indentured laborers in the Caribbean.

## Methods

### Inventory of ancient Asian landraces

From historic literature and labels of rice specimens in the collections of Naturalis Biodiversity Center (L) in Leiden, the Netherlands, we compiled a database of names for Asian rice varieties that were mentioned as being grown by Indian contract laborers and small-scale farmers in Guyana and Suriname between 1868 and the present (Additional file 1). This included traditional names of Indian landraces, and several early rice cultivars developed by agricultural institutes (named after breeding institutes and agronomists) and traditional Javanese, Maroon and Creole landraces (with names in these languages) that were known to be distributed among Hindustani farmers or grown in experimental plots. We searched the Genesys platform (www.genesys-pgr.org) on which online information is stored about crop genetic resources to see if germplasm (living seeds) of these variety names was conserved in gene banks. We photographed specimens of historic varieties kept at in the Naturalis herbarium, and collected a few grains of each variety name that we glued husked and dehusked under transparent tape on white paper. These papers formed a ‘rice book’ that was shown to rice farmers and their descendants in Suriname, used as a prompt for the semi-structured interviews. Throughout this paper, we use the Surinamese (Javanese) term ‘padi’ for husked rice, different from the English term paddy, which indicates a wetland rice field.

### Field interviews

In the Netherlands, we interviewed the managers of two religious Hindu shops in The Hague and Zoetermeer on ritual uses of rice and possible contacts of rice farmers in Suriname. Fieldwork in Suriname was carried out between 27 September and 22 November 2018. Semi-structured interviews were held with (family members of) currently active and former small-scale rice farmers of Indian descend, and people of other ethnicities (Maroon, Creole, Javanese) who were somehow involved in the cultivation of Indian rice varieties or had been active on rice farms owned by Hindustani in the past. Participants were recruited by means of snowball sampling and previous contacts with family members of (former) rice farmers living in the Netherlands. Small-scale farmers were defined has having (had) rice fields smaller than 10 ha. We did not interview employees of the large, industrial rice farms. Following the suggestions of our Hindustani informants on where to find traditional varieties, we also interviewed one Javanese and several Maroon farmers.

Our questionnaire focused on the varieties grown (now or in the past) by small-scale rice entrepreneurs, their local names and meanings, agronomic qualities (e.g., tendence to seed shattering, lodging, susceptibility to pests and diseases), and origin of the seed stock. We also included questions on traditional and current farming methods, cultural and spiritual aspects of rice cultivation, people’s personal memories of rice farming in the past, as well as their motivations to continue or abandon this practice (Additional file 2). All interviews were conducted by the first author and held in Dutch and Sranantongo (Surinamese Creole), or, with the help of translators, in Sarnami (language of mixed Indian origin spoken by Hindustani in Suriname), or in Aucan and Saramaccan (Maroon languages). The interviews with Maroon farmers focused on rice varieties exchanged with Hindustani farmers in the past. Data from interviews were entered in a spreadsheet to analyze responses. A separate spreadsheet was created with rice varieties mentioned during the interviews and their associated information. We verified the spelling of local names mentioned by the interviewees and scientific names using literature [26] and online databases [27, 28].

### Rice collections

Rice seeds and inflorescences were collected from people’s field and kitchens in paper envelopes and deposited at the SNRI/ADRON in Nickerie and Naturalis. Macro-photographs were made from the husked seeds and dehusked grains of all different rice varieties and off-types. Morphological characteristics of each variety (length and width, husk color, presence and length of awns, etc.) were documented and entered in the spreadsheet. A few grains of each variety were germinated on wet tissue paper to obtain fresh leaf material for DNA extraction, to be used for future genetic research. In Paramaribo, the National Herbarium Suriname (BBS) was consulted to verify whether specimens of traditional East Indian rice landraces had been preserved in Suriname. The database and collections of the SNRI/ADRON were consulted to find out for which traditional Asian landraces the germplasm had been safely stored.

## Results and discussion

### Interview participants

**Fig. 1:**
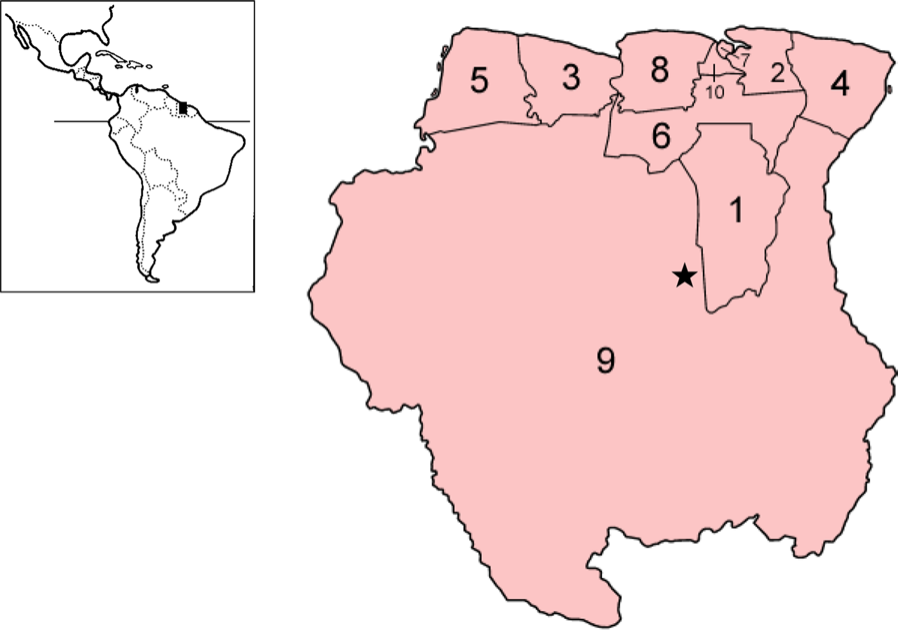
Location of Suriname and districts: 1. Brokopondo; 2. Commewijne; 3. Coronie; 4. Marowijne; 5. Nickerie; 6. Para; 7. Paramaribo; 8. Saramacca; 9. Sipaliwini, 10. Wanica. ★ Jawjaw

In total, we interviewed 41 people (16 men, 25 women), 26 of Hindustani descend, 11 Maroons, one of Javanese origin, and three of mixed ethnicity. Participants resided in the districts of Paramaribo, Wanica, Commewijne, Nickerie, Saramacca, Marowijne and the Maroon village of Jawjaw in the Sipaliwini district (Fig. 1). The Maroon participants (1 man, 10 women) included ten active rice farmers: four Aucans in the villages of Santigron (Wanica district), Tamanredjo and Macreabo km 54 (Commewijne), km 57.5 (Marowijne) and six Saramaccan women and one man involved in the promotion of traditional rice cultivation in Jawjaw. The 30 non-Maroon participants were all somehow involved in Hindustani rice farming: four were current vendors of padi (two in the Netherlands, two in Suriname), five were active rice cultivators, ten were former traditional rice farmers, nine used to work on the rice farms of their Hindustani family or friends, one used to operate a rice mill, one was a staff member of the Lalla Rookh museum on the history of East Indian immigration in Paramaribo and one was the director of the Nickerie department of the Ministry of Agriculture, Livestock and Fisheries (LVV). The age of the participants varied between 28 and 85, with an average of 61 years. Apart from rice farming, they also had off-farm jobs. During the interviews, some participants were joined by other household members, so a single interview often represented the knowledge of more than one person.

### Historic rice landraces

Data on landraces cultivated by farmers of Asian descent in the Guianas is limited and scattered throughout historical agricultural reports, without references to herbarium or seed collections [8, 10, 11, 13, 15]. Still, we retrieved information on c. 16 rice varieties, with in total 24 vernacular names, as some had names in Sranantongo and Sarnami (Additional file 1). Although the Naturalis herbarium has the largest collection of specimens from the Guianas worldwide, no herbarium vouchers or seed material were found for the historic landraces Garika Suama Vari and Kristna Kata Kulu, recorded only for Guyana [10] and Fini tere, first mentioned for Suriname in 1871 [13] and last in 1962 [3]. None of the interviewees mentioned these landrace names, so the varieties have either been lost or are still cultivated under other names. For the landraces Anaki tapoen, Mutmuria, Ramcajara and Sutra dhân, no herbarium material could be located. Of these four landraces, farmers only remembered Mutmuria, which was said to be still grown occasionally in the Saramacca district for both human consumption and chicken feed. The available germplasm for Mutmuria also originated from Suriname (Additional file 1), while rice varieties with similar names (Mutmuri, Mutmiori, Mutmurda) have been documented in India between 1878 and 1909 [29]. Several of the rice cultivars that were planted in the experimental station in Paramaribo had been preserved as bundles of panicles wrapped in paper or stored in wooden boxes in the Economic Botany collection of Naturalis (e.g., Padi Nickerie, Blue Rose and Demerara Creole). Farmers still remembered several of these historic cultivars, such as Padi Nickerie, Padi Tjina, Blue Rose and Demerara Creole, of which the latter was still said to be cultivated, but no details of the location were known, so no seeds could be collected. Although most of the Hindustani we interviewed had abandoned rice farming, they still mentioned many varieties that they had grown in the past or had seen on other people’s fields. We compiled a list of 55 variety names, with information on their agronomically and culinary characteristics, origin, former and current collection localities (Additional file 3). Of these 55 names, nine were also mentioned in the historic literature (Additional file 1). We were able to collect only four different varieties on rice fields of Hindustani farmers. The Maroon farmers mentioned c. 18 additional names for rice varieties, 10 of which were collected.

The most widely known historic landrace from Suriname is Skrifimankoti (also spelled as Srivimankoti), that translates as ‘writer’s coat.’. Imported in 1890 by East Indian immigrants, it became the most important wetland variety in Suriname, suitable for the heavy clay soils in the coastal plains [13, 30]. By 1904 it was the best performing variety and was still grown by smallholders in Suriname in the early 1960s [3, 15]. Skrifimankoti was unsuitable for mechanized harvesting because of its tall stems and tendency to lodge [15, 30]. It had a growth season of six months, and a high value on the global market due to its white, elongated and thin grains [3, 15]. Skrifimankoti was considered to be the same as the Indian variety Patarka dhan [30], and quite similar to the Demerara Creole from Guyana. Observers also compared it to Carolina Gold from the US. In the 1900s, seeds were sent to breeding stations in the US and Java, where they were distributed to farmers [31].

Of the 30 interviewees that were (once) involved in Hindustani rice farming, ten remembered Skrifimankoti. They confirmed that it took more than five months to ripen, but the quality of the rice was good and the yield was high. They remembered it as the ‘miracle variety’, but it lodged easily, especially when not harvested on time. Respondents said Patarka dhan (‘thin rice’) differed from Skrifimankoti in that it ripened in four months, had a higher yield, nutritious grains, and a good resistance to pests and diseases. It lodged easily and was harvested by hand with a sickle. Demerara Creole had shorter grains than Skrifimankoti and came from Guyana. This was confirmed by the historic seed collections from 1932 in Naturalis, of which ‘Padi Demerara’ (L.2110084), had brown-tipped grains that were somewhat shorter than ‘Skrivimankoti’ (L.2110089). Living seeds of Skrifimankoti are preserved at the SNRI/ADRON germplasm bank and represent one of the few Indian rice landraces from the Guianas that are still preserved today (Additional file 1).

Most respondents knew the difference between old landraces and modern cultivars. As former rice farmer Mrs. Chitrawatie (60, from Saramacca) explained: “You could recognize old rice landraces by their height: they were often 1.5-m tall, the modern cultivars are shorter. Nobody grows these old races anymore I think […].” The farmers remembered that the high plants lodged easily when it was almost time to harvest, which resulted in a loss of yield.

Landraces that were said to be grown before the mechanization and harvested and peeled by hand were the sticky Javanese Ketan varieties, Shifonia, Barbaman and Padi oeloe (Additional file 3). Typically, some Hindustani farmers also cultivated Ketan rice in the past. The yield was exchanged with Javanese women, who would process it into sweet rice dishes for the Hindustani families. Barbaman (‘bearded man’) and Padi oeloe (‘worm rice’) had long awns. Awns protect grasses from predation by animals, and aid seed dispersal by clinging to animal fur [32]. They are also a nuisance during mechanical seed processing and storage, so from the 1960s, modern rice cultivars in Suriname were awnless [15].

**Fig. 2:**
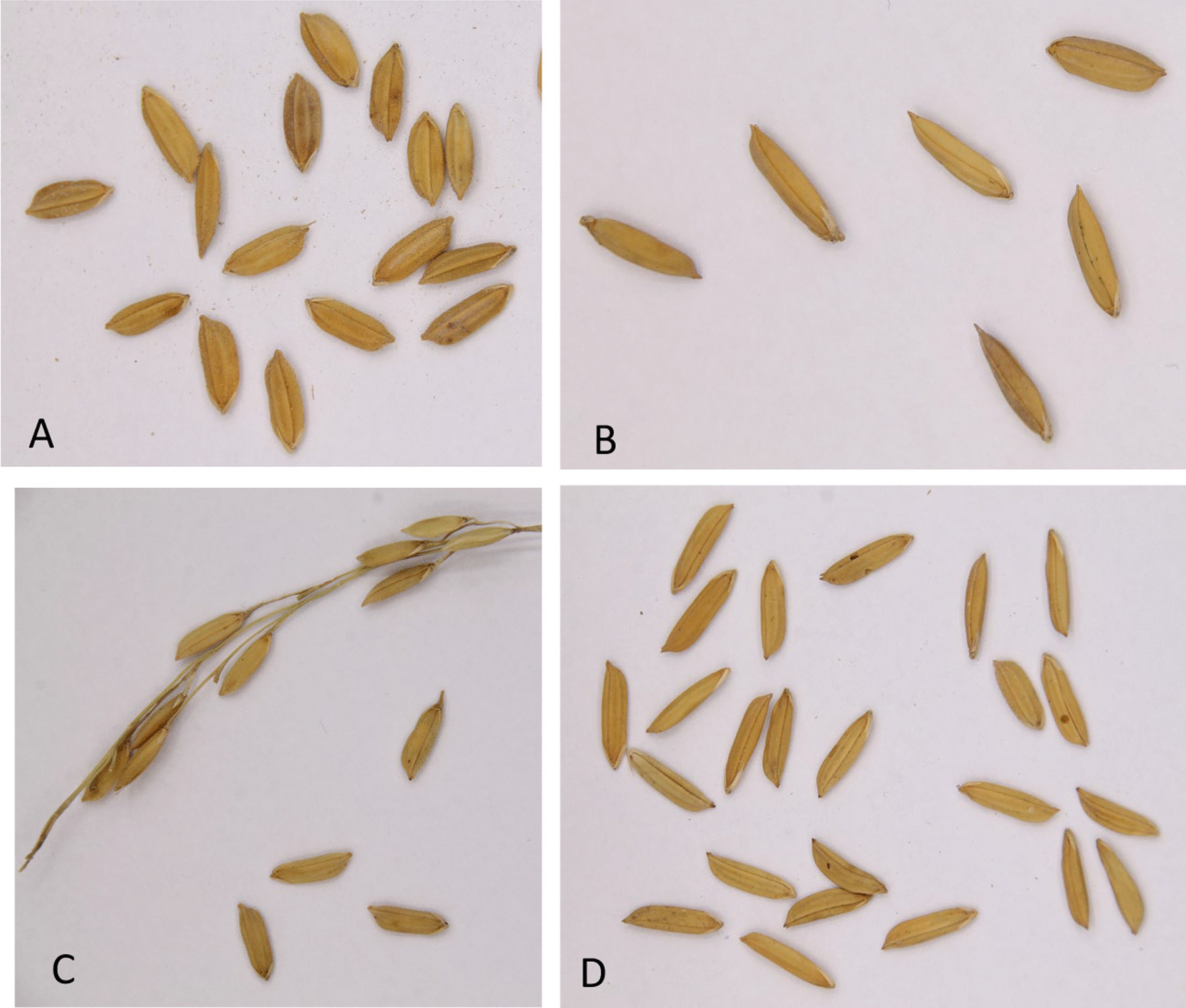
**a** Raymoen (MMR6) showing variability of grain sizes. **b** White Sola (MMR11) grown by an Aucan farmer in Macreabo. **c** Dima (MMR18), collected in Marowijne, Km 47. **d** DDB2 (MMR5), grown in Saramacca. Pictures by Micha de Boer

In 2018, the only landrace that was still cultivated by our participants was Raymoen (Fig. 2a), grown by one farmer in Saramacca (Additional file 3). Also known as Ramoen or Ramona, it was probably a landrace of Javanese origin, with tall stems (c. 1.5 m), ‘good, nutritious padi, soft when cooked’, and no agrochemicals were needed. The negative aspects of Raymoen were the tendency to lodge, the variability of the grains, and low yields with ‘hard seeds’ when there was wind and rain during harvest. It was used for home consumption and chicken feed. Typically, one farmer said it was ‘the same as Mutmuria’, but as we have no specimens for the latter landrace, morphological comparison was impossible.

### Historic and modern cultivars

The best-known historic cultivar (remembered by 14 Hindustani and the single Javanese participant) was Rexora, also known as Bergi (hill) Lexora (Fig. 2b). This non-lodging, long-grained upland cultivar was imported in the spring of 1932 from Louisiana (US) and planted in experimental trials in Paramaribo to see whether it was suitable to replace Skrifimankoti on poor, better drained soils [33]. In 1936, a bag of Rexora was sent out to the Saramaccan Maroon village of Ganzee [30]. Although none of the Hindustani (former) farmers we interviewed still cultivated it, many of them praised this cultivar for its high yield, good taste, nutritious value and resistance against diseases: it did not need any pesticides and thrived on drier grounds. The Rexora cultivar was abandoned by commercial rice farms in the 1960s after a severe attack of Cercospora disease [34]. However, the Aucan women we interviewed in the Commewijne and Marowijne district and the Saramaccan women in the Sipaliwini district still actively cultivated an elongated upland rice variety that was known as Alekisola or White Sola. This ‘(Aleki-) sola, genetically quite distinct from traditional Maroon landraces [35], is likely to be the locally adapted progeny of the 1960 cultivar Rexora. It was also grown by Aucans along the Marowijne and Lawa rivers in 2017 [35] and along the Tapanahoni river in 2013 (Naturalis L.3928210). The ‘Red Sola’ rice represents different Maroon landraces that are genetically distant to Rexora [35].

Several of the other 43 varieties named during our interviews referred to old cultivars that were said to be ‘introduced by the Dutch’ after the beginning of mechanized rice farming. Examples are Alupi, Apikalo, 81B and Holland (Additional file 3), developed by SML in the 1950s and 1960s [7]. SML cultivars developed from the 1970s onwards often received names ending with -ni (after Nickerie), the seat of the breeding institute. The farmers had mixed feelings about the qualities of these early cultivars: some had ‘good cooking quality’ (Diwani, Alupi, Eloni, Dima, Holland), but others were lodging (Campochino, Holland, 81B), shattering (Dima) or remained hard when cooked (Camponi).

Fifteen (former) Hindustani farmers said Dima was a good cultivar, which was still occasionally cultivated in the polders of Wageningen (Nickerie district). Developed from Skrifimankoti in 1953 for mechanized harvesting and named after the rice breeding pioneers Van **Di**jk and **Ma**stenbroek, Dima had stiff straw to prevent lodging, and was considered far superior than other popular cultivars at that time, such as Rexora, Bluebonnet and Skrivimankoti itself [3, 15]. We collected specimens of Dima from a 66 year-old Aucan Maroon farmer along the East–West highway in the Marowijne district (Fig. 2c). She said that Dima was also planted by her grandparents, who had obtained it from Hindustani farmers. In spite of its shattering panicles, this knee-high cultivar with elongated grains produced sweet rice when harvested and peeled by hand. Around 2005, ethnobotanist Bruce Hoffman (pers. comm.) also heard about a rice variety named Dima in Stonhuku, a Saramaccan village along the Gran Rio, deep in the interior of Suriname. An Aucan farmer in Marowijne said she previously also planted the 1963 cultivar Hollandia, which thrived on low and wet soil, just like Dima.

At least ten rice names mentioned during our interviews referred to (relatively) modern cultivars, with short growth seasons and many seeds per panicle, developed after 1995 by SNRI/ADRON or commercial companies like Manglie, Ini-Dia and Paloma. According to the farmers, the name of the cultivars referred to the number of days the plant took to ripen. The once popular cultivar 110, which had a heavy panicle with thick, long and yellowish seeds, and took 110 days from sowing to harvest, was said to have replaced Skrifimankoti. The cultivar DDB2 was developed in 2017 by SNRI/ADRON and named after Suriname’s controversial ex-president Desi Delano Bouterse (Fig. 2d). According to our participants, DDB2 was susceptible to diseases, had weak stems that lodged easily, and implied high costs and little profit, which was confirmed by SNRI/ADRON [36]. It was still grown by one of our participants in the Saramacca district for home consumption and the market. The modern cultivar 125 was grown in large quantities in Nickerie, but was also susceptible to fungi [37]. The farmers we interviewed never used the latest cultivars developed by the SNRI/ADRON around 2010, such as Elitezaad ADRON-128.

### Seed sources

Our respondents knew from their family history that the indentured labourers from India brought their own rice landraces, kept some of their harvested rice as seed stock to plant later on, and traded or bartered planting material within the Hindustani community. The different ethnicities (Hindustani, Javanese, Creoles, Maroons) lived (and still live) in separate areas, while seed swaps were mostly done within the neighbourhood. Exchange in rice with other ethnic groups were therefore rare events, but did take place occasionally, as indicated in the previous section. From the 1930s onwards, the breeding institutes (SML and later SNRI/ADRON) started to distribute improved cultivars among farmers, and discouraged them to use their own seed stocks for sowing the next harvest. Still, several farmers used their homegrown rice as plant material, or experimented with unknown varieties obtained from other farmers. A 71 year-old farmer recalled that as a young girl she picked up seeds that fell on the street from a load carried by a man passing by. Her father planted them in his field in the Henaerpolder (Nickerie district) and it produced a very short rice plant, with a full, pale brown panicle and good taste.. This rice type, probably a commercial cultivar, was grown for years by this family and named Doerdjie (possibly referring to the Sarnami term for semolina (soedjie), see Additional file 4).

In recent decades, the Ministry of Agriculture and Fisheries (LVV) and SNRI/ADRON have continued their efforts to convince farmers to buy registered seed of modern cultivars [38]. Commercial rice production for export is protected by a national law that prohibits the use of seed from your own planting or buying ‘illegal’ seed, not controlled by LVV or SNRI/ADRON [38]. However, some of the (former) rice farmers we interviewed said that in spite of their lower quality, rice cultivars from Guyana were becoming more popular, as they needed less agrochemicals. This suggests the existence of informal seed exchange networks, although we did not study these in detail.

### Weedy rice

**Fig. 3:**
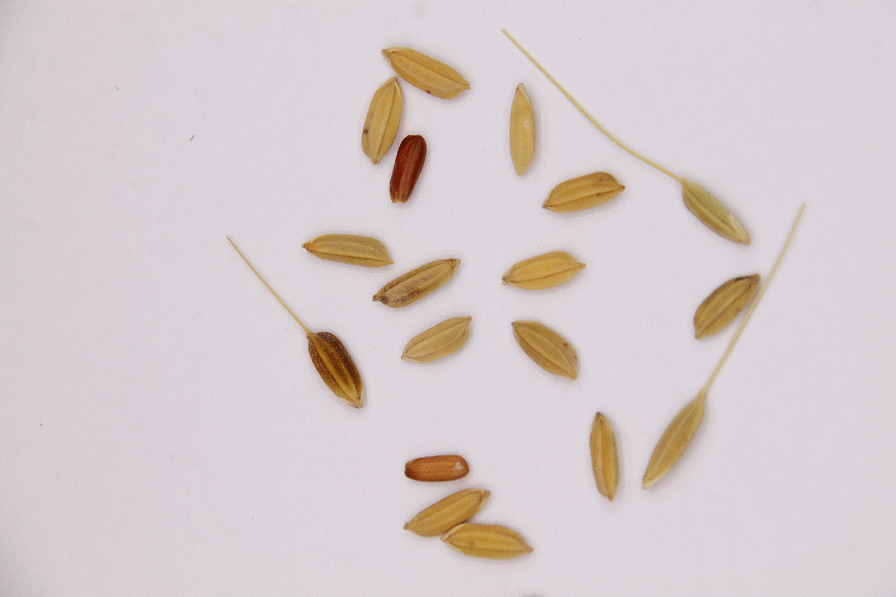
‘Nickerie padi’ fodder rice sample (MMR3) mixed with different types of weedy rice (awned and hairy husks, red seeds). Picture by Micha de Boer

Our sample of ‘Nickerie padi’, grown for fodder, contained grains that varied substantially in size, shape, colour and the presence of hairs and awns. When milled, a proportion of the bran was red. This fodder rice consisted of long-grain (domesticated) rice, mixed with weedy rice, which are hybrids between *Oryza sativa* with wild rice (*O. rufipogon* Griff.), characterized by hairy and ridged husks, awns and red bran (Fig. 3). The fodder rice also contained seeds from true rice weeds, such as *Ischaemum rugosum* Salisb*.* with inflorescences that looked like the segmented insect bodies. Hindustani farmers called the weedy rice ‘lalya’ (red in Sarnami) and recognized it in vegetative form by its tall stems and broad leaves.. Weedy rice is quite hard to get rid of, since the seeds survive in the wet clay soils for years. According to our participants, weedy rice was a greater problem in wet rice fields and mechanical rice farming systems than it was before in manual rice cultivation. All Hindustani farmers we interviewed encountered weedy rice on their field, and farmers who only grew rice for fodder (mostly chicken feed) were not bothered by it. One farmer used to plant a six-month upland variety named Moeng that was also a mixture of grains with red and white bran. This fodder rice, sold for c. €5 per 10 kg in Suriname, facilitates the spread of rice weeds in the coastal plains. Remarkably, some of this mixed rice ends up in ritual Hindu shops the Netherlands, where it is sold for a much higher price (€ 4,95 for c. 250 g) to be used during religious ceremonies (MMR1, Additional file 3).

### Hindustani rice on Maroon fields

**Fig. 4:**
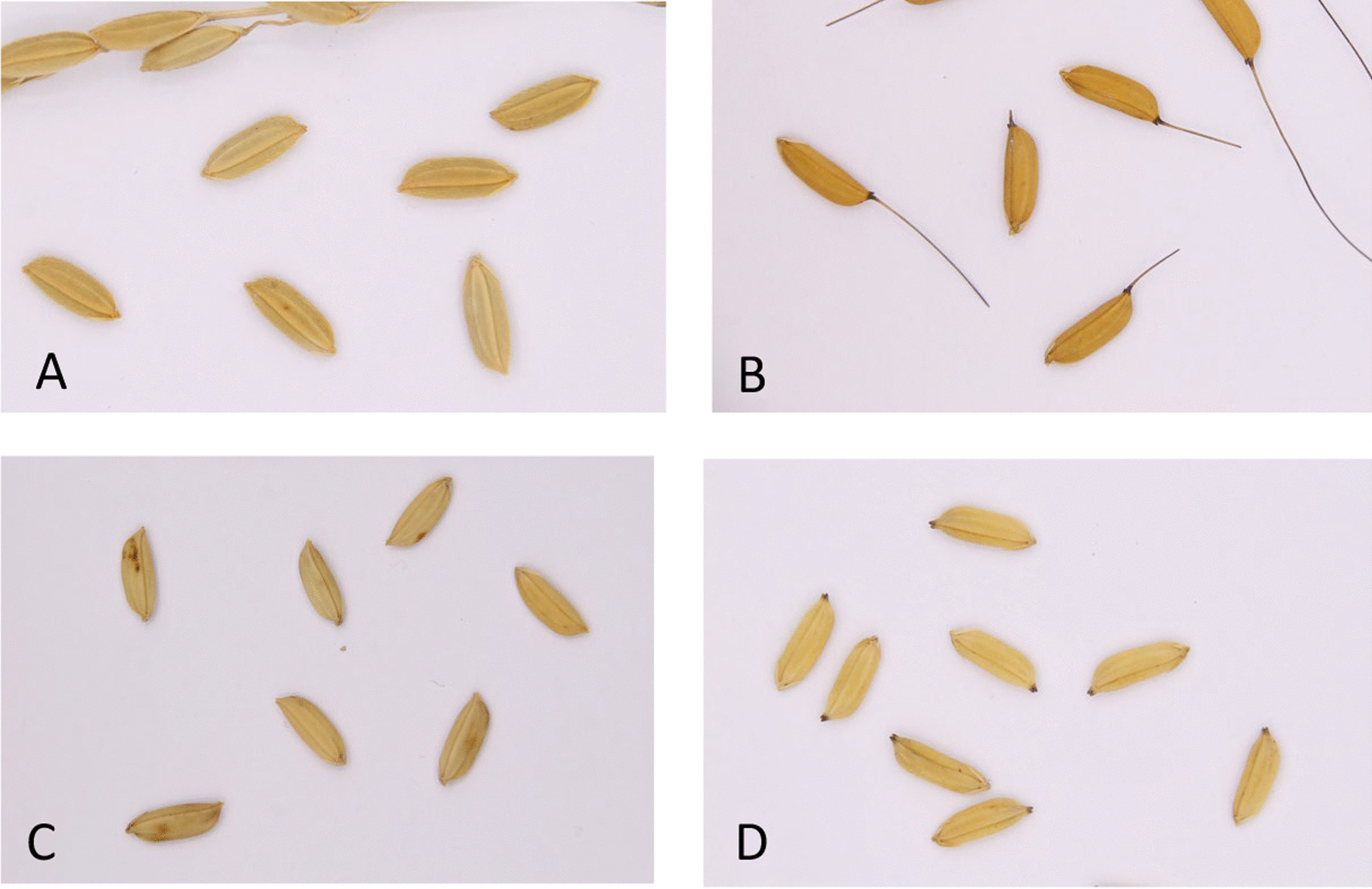
**a** Koelie aleisi with hairy husks (MMR31); **b** Koelie aleisi with awns and glabrous, orange husks (MMR30); **c** Koelie aleisi with glabrous, white husks (MMR29); **d** Watralanti (MMR34). All varieties were grown by Saramaccan Maroons in Jawjaw. Pictures: Micha de Boer

Saramaccan Maroon rice farmers in Jawjaw cultivated several varieties named Koelie aleisi (‘coolie rice’), which differed in husk color, pubescence and presence of awns (Fig. 4a–c). Some Maroon women said that these varieties were traded by their grandparents with Hindustani people or bought in the city several generations ago. Another variety of possible Indian origin was Watralanti (‘waterland’), which had to be planted on lower, moist ground, just like the Koelie aleisi, which suggests both descend from wetland varieties (Fig. 4d). One interviewee said that she had ‘seen Watralanti rice herself being planted by Hindustani farmers in Nickerie’. Herbarium collections of Watralanti rice were collected from Maroon rice fields for an earlier study [35], see Additional file 3. Baumgart et al. [39] collected a Watralanti variety in 1998 in the Saramaccan village Nieuw Aurora, which also had the name Jarikaba (DMR 980029), which refers to a region in the coastal Saramacca district with East Indian and Javanese farmers. They also recorded the varieties Mboto Molia (DMR 980011) and Mbotomolia or Botombolie (DMR 980018) in Nieuw Aurora and suggested that these varieties descended from traditional Hindustani rice types, in particular the landrace Mutmuria [39]. In 2005, a variety named Patalika (which resembles ‘Patarka’) was recorded by Bruce Hoffman, (pers. comm.) in the Saramaccan Maroon village Stonhuku (Gran Rio), but unfortunately no collections or photographs were made.

In a recent molecular study, a single accession of Watralanti from the Marowijne area was shown to be genetically quite distant from traditional Maroon landraces, but quite similar to the SML cultivars Ciwini and Acorni, developed in the 1970s [35]. This suggests that some of the ‘coolie rice’ grown by Maroons may descend from improved cultivars developed after the 1960s, instead of from traditional Indian landraces. The Aucan farmers who we interviewed in Commewijne were in regular contact with Hindustani rice growers and also grew cultivars developed after 1960.

Buying seed stock is exceptional among Maroon rice farmers, as most landraces are handed down within the household from generation to generation or exchanged with extended family members during funerals [35, 40]. The potential presence of East Indian rice varieties on Maroon farms indicates that occasional introductions of ‘non-Maroon’ rice varieties have taken place in the past, and in some cases were successful. Trying out new rice types, however, is not something of the past. Andelia Boenakaba, a 65 year-old Aucan rice farmer from Macreabo (Commewijne) recognized the historic cultivar ‘Padi Nickerie Wageningen’ from our rice sample book. She said she once bought it at the central market in Paramaribo and sowed it in her field. A Saramaccan farmer in Jawjaw also said she bought husked padi at the same market, and was surprised to see that it grew into a dwarf rice plant. Our research indicates that both historic landraces and modern wetland cultivars have found their way to Maroon rice farms in the interior, facilitated by markets and experiments of curious farmers.

The historic rice exchange between Hindustani and Maroon farmers could be confirmed by additional collection of herbarium specimens from Maroon rice fields and molecular research, in which the DNA of ‘coolie rice’ types is compared to that of historic herbarium samples and existing genomic data on East Indian varieties kept in germplasm banks. This can shed light on the geographic origin of these Asian rice cultivars and fill gaps in the history and provenance of Indian contract laborers. This is not only relevant for the agricultural history of Hindustan in Suriname, but also for those in Guyana, who lost most of their traditional landraces already in the early 1900s [9].

Very little information exists on traditional rice cultivation by Javanese farmers in Suriname today [23]. Molecular comparison of the many Javanese rice accessions from the 1930s in the Naturalis collection and landraces currently grown in Suriname could reveal hidden information on the migration of Javanese people, and the adaptation or their crops to a new environment.

### Current and past motivations for rice cultivation

The main reason for our interviewees to cultivate rice was home consumption: 17 of the 30 Hindustani participants had cultivated rice by hand for more than 30 years. When they were young, there were few other job opportunities outside agriculture. Before the development of mechanical rice farming, their families did not have enough money to buy rice in a store, and it was not even common that shops sold rice. It was essential to be self-sufficient and grow your own food. Rice was and still is the basis of many dishes eaten for breakfast, lunch and dinner. In the past, some people used home-grown, hand-milled rice to barter for products in grocery shops. Former rice farmer Mrs. Chotkanoe-Jhinkoe (70) from Wanica remembered that she traded “one kilogram of rice for one kilogram of salt or sugar at our local store”. If there was more padi in stock than needed for home consumption, the surplus was stored in a shed behind the house and sold in the husk to merchants. With the money earned, the farmers could buy other provisions. “We sold one bale of padi for three Surinamese dollars. In those times that was a lot, so it was very valuable”, said Mrs. Hondoe (80) from Nickerie.

The by-products of rice production, such as husks, bran and broken grains were and still are used as fodder. Unmilled padi is also fed to chicken and ducks. The milky juice squeezed from unripe rice panicles was said to be very nutritious for songbirds, popular pets in Suriname, and experimental fields of SNRI/ADRON were often raided for young inflorescences by songbird traders [41]. Pig feed consisted of husked padi, ground rice flour, waste material and broken grains. Leftover cooked rice was fed to the dogs. Farmers warned not to feed rice products to cows, as this harmed their stomachs. While most people used the same rice variety for human and animal consumption, others cultivated short grain cultivars for fodder and kept the long grain types for their own consumption or for sale. Short grain rice did not sell for a good price, but had a higher yield per plant, which made it favorable for feeding livestock. All parts of the rice plant were put to something useful in the past. Mrs. Ramadin (71) from Nickerie recalled that when she was young the rice straw was used to construct roofs and walls of the little huts in which people lived.

After the modernization of the rice farming, many traditional Hindustani rice farmers could not afford the investments in machinery and were unable to compete with the large-scale producers. They sold their plot of land and moved to the city, where their children enjoyed better and longer schooling than their parents and grandparents. The highly educated new generation was eager to find better paid employment outside agriculture, and after Suriname’s independence in 1975, many of them migrated to the Netherlands. Those traditional farmers who also grew vegetables and fruits decided to abandon rice, since they could make more profit with garden produce. The remaining small-scale rice farmers currently all use tractors and other machinery, but they can still hardly compete with large-scale rice producers. According to Mr. Harimandan, chairman of the Surinamese padi farmers association (SPBA), Guyana is currently taking over the rice market. Many rice farmers search for better job opportunities to escape the financial risks that they face. The plummeting market prices for padi lead to a rapid decline in the number of small farmers and a decreasing quality of the product. Also, the lack of maintenance of roads and draining channels by the municipalities makes access to rice fields difficult, and contaminated water is not being refreshed [42].

**Fig. 5:**
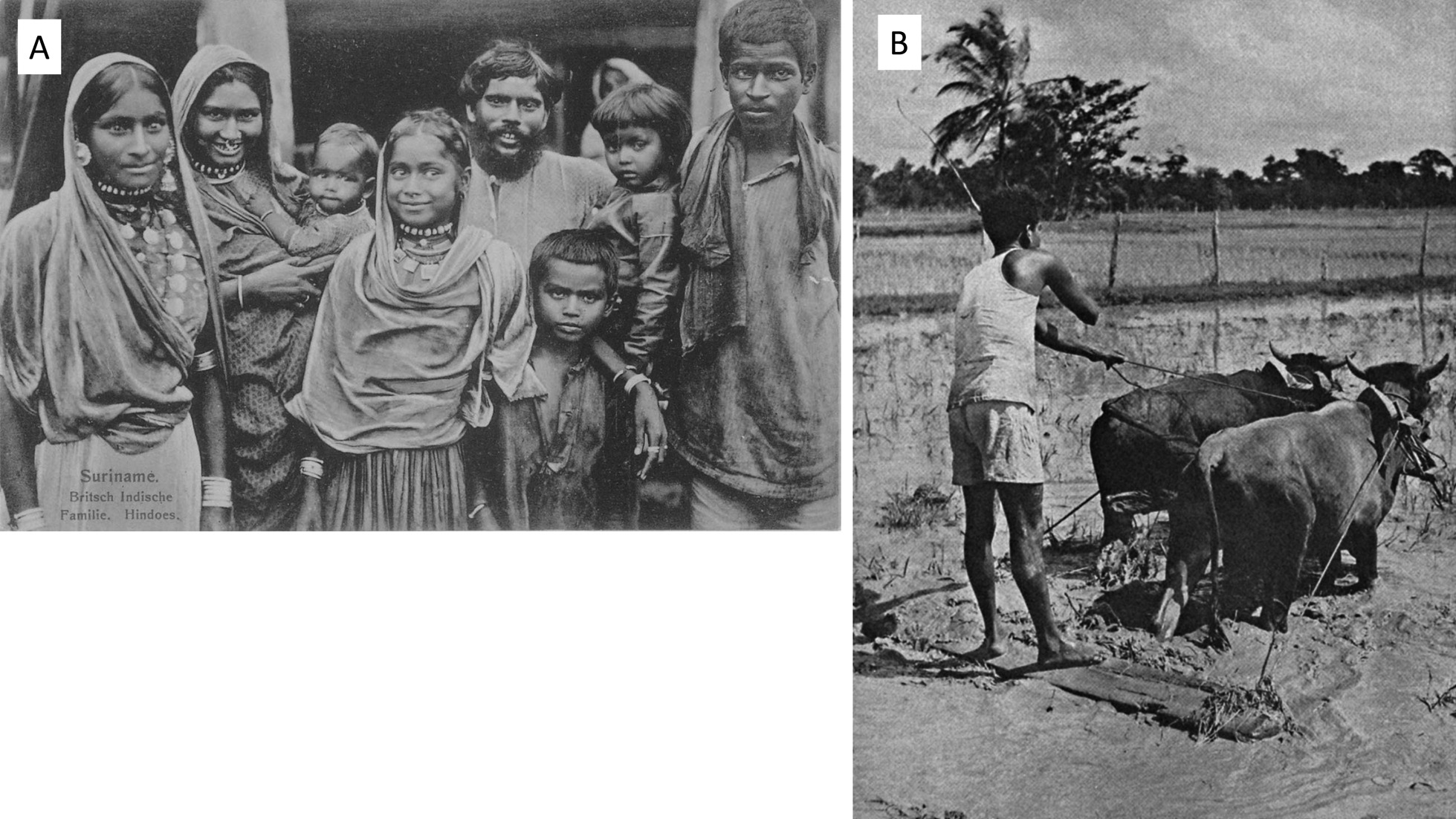
**a** First generation of Indian indentured laborers in Suriname (c. 1900): the forefathers of the current Hindustani rice farmers. Picture: Museum of World Cultures (1900-A296-51); **b** Hindustani farmer levelling a rice field with two bullocks, Suriname (1905). Picture: University of Amsterdam special collections (URI01-2792H39PL122)

While the modern rice farms expanded, some Hindustani smallholders continued with traditional rice production until they became too old and there was nobody left or willing to help them on their fields. Several elders we interviewed would have loved to maintain their rice fields but were unable to do so. Mrs. Bhoelai (62) from Houttuin, now a vegetable vendor at the Central Market in Paramaribo, explained: “We became too old and our children did not want to help us, as they found it hard and dirty work. I would have liked to continue [rice cultivation], since I was brought up with it and was used to it”. Thinking of their farming days and their own rice fields, they felt a sense of happiness and pride. “My ancestors did it this way [by hand], I had their blessings if I also did it like that”, explained Mrs. Jambadjan-Bhawari (88) from Nickerie. Once their children moved out of the house, the need to grow rice for home consumption also disappeared. The few small to middle-scale commercial rice farmers that remained active said they were ‘born in the rice business’. Their fathers or grandfathers had invested in good farmland and machinery and they felt that if they would quit rice farming, these investments would have been lost. Mr. Soewad (40) a small rice farmer in Nickerie, spent his spare time working on his rice field, when he was free from his regular job as a taxi driver. “For me, rice farming is a hobby. I love doing it, it makes me feel relaxed. I took over the land and machines from my father, so it’s in my blood, I must do it”. For both the current and former farmers we interviewed, rice cultivation was more than a cheap way of obtaining food or a necessary job. It was also a source of pride and joy and a way of honoring and being spiritually blessed by their forefathers (Fig. 5a).

### Division of labor

**Fig. 6:**
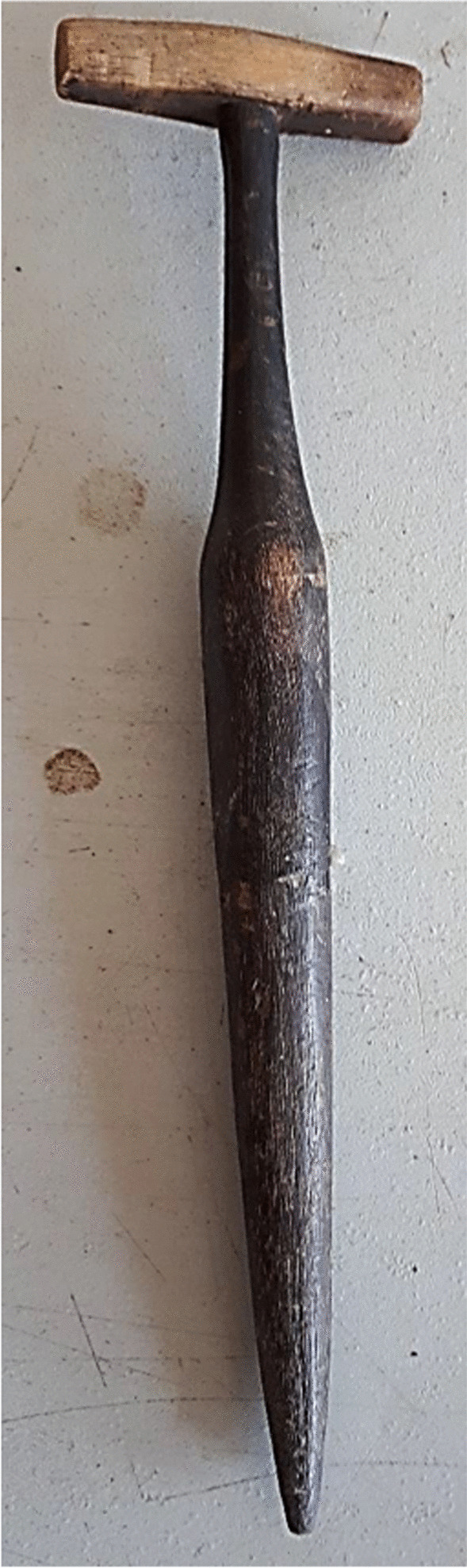
Traditional rice planting stick (kuthi) owned by antiquarian in Nickerie. Picture: Melissa Ramdayal

In traditional rice farming, there was a division of labour between gender and age groups. Overall, men were involved in sowing the padi in the submerged clay soils, plowing the fields with bullocks (Fig. 5b), threshing the panicles and using heavy tools. Mrs. Ghowrising (61) from Utrecht, the Netherlands explained: “women planted the bibit, by hand or with a planting stick (Fig. 6) and weeded the fields, but during the harvesting period, many hands were needed and entire families worked together”. Women generally worked on the rice fields between 7 am and 5 pm, while their oldest daughter or the grandparents would take care of the smaller children. Former rice farmer Mrs. Hondoe (80) explained: “Every family had many children. My family had eight, and my aunt had 12. We were neighbors, so we were very close and lived and ate together on a daily basis. As a woman you raised a total of almost 22 children from the whole family: your own, those of your brothers and those of your husband's previous wife”.

**Fig. 7:**
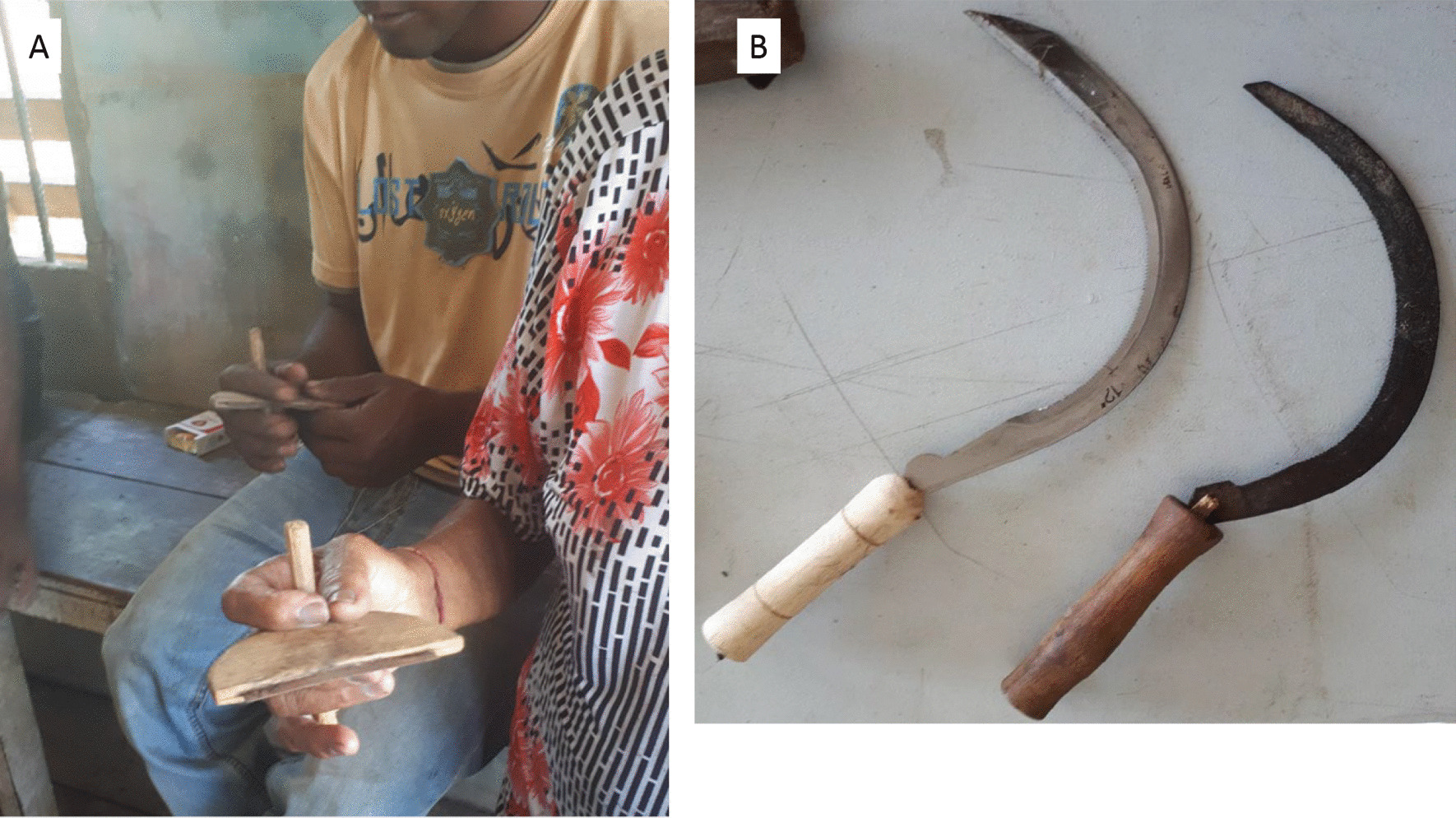
**a** Former rice farmers demonstrating how to hold the ani ani, a traditional Javanese harvesting knife. **b** Modern (left) and antique (right) haswa. Pictures: Melissa Ramdayal

**Fig. 8:**
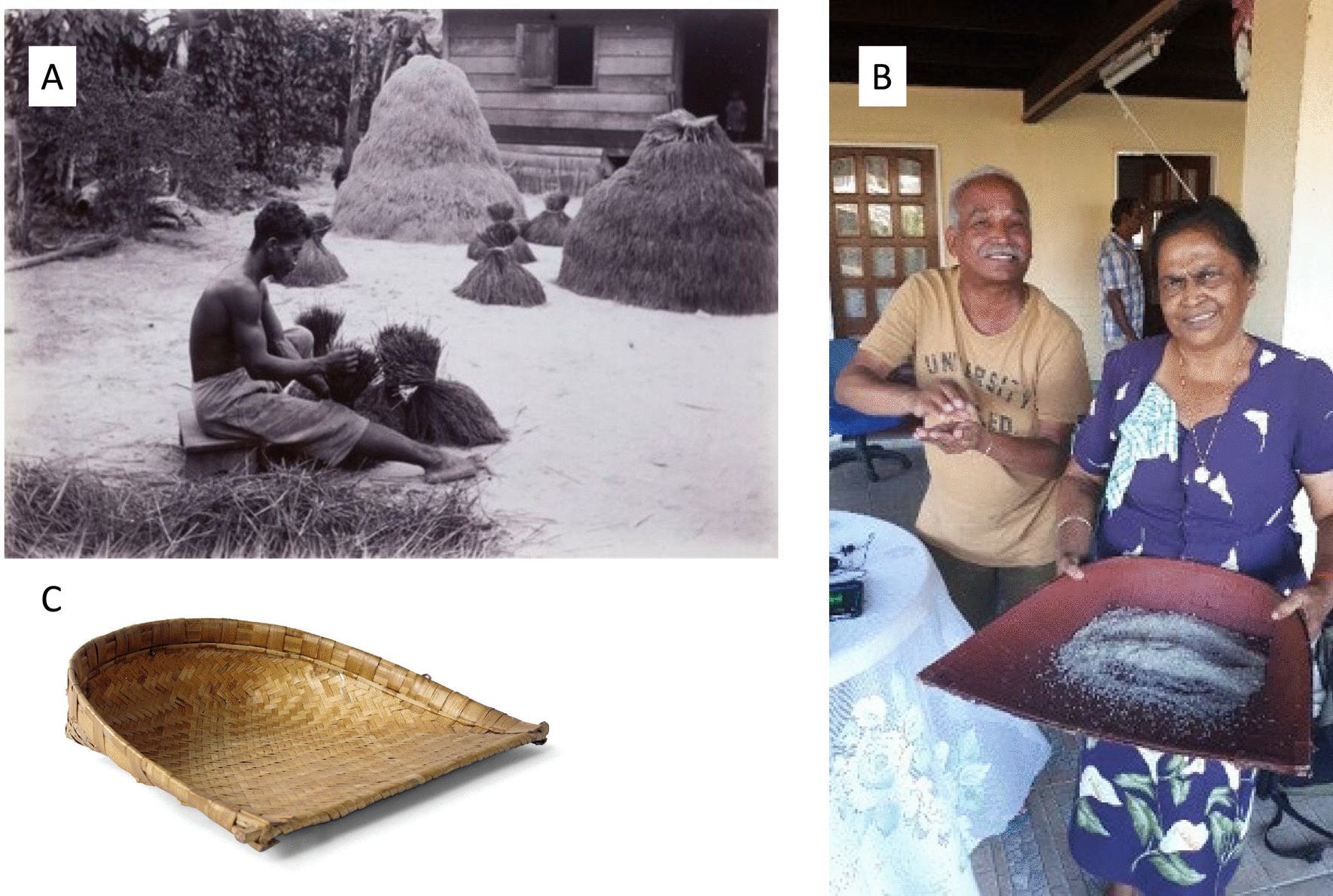
**a** Farmer tying bundles of harvested rice. The mounts of rice panicles are visible in the background, Suriname (c. 1920); **b** Mrs. Ramadin from Nickerie showing a traditional winnowing fan (‘soep’); **c** Traditional Indian winnowing fan. Pictures: A. Museum of World Cultures (TM-60006030); B. Melissa Ramdayal, C. Odisha State Tribal Museum, Bhubaneshwar, India

Most children began helping in the rice fields at the age of 14, starting with light tasks, like carrying the harvested panicles out of the field. All harvesting was done by hand, using traditional tools, such as the ani ani (Fig. 7a) and the haswa (Fig. 7b). Rice was stored in sheds (Fig. 8a) and winnowing was done with a woven winnowing fan (Fig. 8b), resemblingthe traditional model still used in India (Fig. 8c). From 1878 onwards children of immigrants were obliged to attend school from the age of seven to 12, but many of them skipped classes during the busy planting and harvesting periods. “Normally, children were only allowed to help when they were finished with their school and homework”, said Mrs. Oemawaty (77). These practices from an important mechanism by which knowledge about varieties and crop cultivation skills are transferred between generations. “When you had 25 people working on your rice field that meant you'd be working for a total of 25 days for the people that helped you. This was a common practice among both Hindustani and Javanese farmers, known as ‘ikta’ in Sarnami and ‘samhatan’ in Javanese, both meaning ‘unity’, explained Mr. Harimandan (78), chairman of the Surinamese padi farmers association.

Rice has played and still plays an important role in Hindu rituals and religious practices. Twelve of our interviewees were Hindu, one was Muslim and five were Christian. In the past, Hindustani farmers carried out a ritual to ensure a prosperous harvest by gathering a bunch of unripe rice culms, removing the young grains by hand and cook them with milk, sugar and ghee (clarified butter). They prayed on the rice field and went home to consume the blessed sweet rice dish (‘persawt’). All interviewees agreed that the rice used in Hindu rituals did not necessarily has to be self-cultivated. In former times people used their home-grown padi, but now they bought rice for their rituals from farmers, on the market or in ritual stores.

### Ritual practices

Rice symbolizes prosperity, welfare and fertility in wedding rituals, as a good harvest means lots of rice, food and income. A Hindu wedding ceremony is held in a small tent (‘maaro’), in which different rituals are carried out. Husked rice is fried like popcorn and stirred with a broom made of coconut leaf midribs (‘printa sibi’) in a pan (‘karhaija’) over a wood fire by the aunts of the bride and groom. It is important that the padi is dry, otherwise it will not pop. This ritual food is known as ‘laawa bhoedjaawe’ (puffed rice), and for this reason husked rice is sold in ritual Hindu shops in the Hague. During wedding traditions of the Sanatana Dharma movement, the bride (‘dulhin’) and groom (‘dulha’) smear cow dung in a certain pattern in an earthenware, brass or copper bowl (‘talsa’ or ‘lota’) when they arrive in the bridal tent. The bowl is decorated by pressing padi in rows in the cow dung. When the bride leaves her parental home and is symbolically given away to the husband’s family, her mother throws padi on her from behind to ensure the newlyweds stay happy. During funerals, padi is scattered on a wooden board, covered with sand and then laid down in running water. Most wedding and funeral rituals are led by a pandit. Hindus from the Aria Dewaker movement give the pandit a 10 or 15-kilo bag of milled rice after the rituals are performed, while the Sanatana Dharma normally pay the pandit a considerable sum of money.

To celebrate Diwali, the festival of light, Surinamese Hindus harvested some handfuls of rice from their field—whether it was ripe or not—and cooked the sweet rice dish ‘mita bhaat’ with sugar and milk. In some religious ceremonies, padi was offered in running water. In the past, people also made ‘rice roti’: a flat bread made from flour of uncooked, pounded rice that was sieved, mixed with salt and baking soda and pressed flat with the hands. The dough was baked in a hot pan with oil and turned until it was baked on both sides. Because of the large-scale import of wheat flour, the laborious rice roti is not often made anymore.

Evil spirits are considered to hide in the rice fields and nearby forests. Mrs. Karmale (66) from Mariënburg carried garlic or a pin with her when working on her rice field for protection. It was not a common practice to work on the rice field in the evening. Former rice farmer Mrs. Hondoe: “After six o’ clock you cannot work on field. Hindustani believe that everything is resting and the plants are sleeping, so you cannot interrupt them. Dark devilish things wake up after that time. There used to be no light after six, so it was dark and people were afraid of everything and went home.” The Sewbaran family remembered that some people used a ‘tapu’, a powerful amulet to protect themselves against evil powers. “When using this aid, it was not permitted to look at a pregnant woman, as she would have a miscarriage.” Mrs. Karmale also remembered rituals to protect babies and mothers, such as a pin attached above the front door or a drink that contained shavings of deer antlers.

Former rice farmer Mrs. Bhoelai (62) from Houttuin explained: “On almost every property, there is a part that is said to be haunted, because long ago it used to be an Amerindian burial place. If you disrespect the soil there, for instance by washing dirty clothes, this could lead to misfortune, miscarriage or even the death of yourself or a loved one. I don’t believe in it, but my mother-in-law does.” Younger generations said not to not believe anymore in these ghost stories, but were still fascinated by them, while older generations either refused to talk about them or denied they knew anything about them. Still, some people would not walk on certain parts of their property. Both Hindustani and Maroon farmers believed that dark-husked rice varieties would have lighter husks when they were sown during full moon, and some planted darker rice types on purpose during this period.

#### Indentured labor and crop diversity elsewhere in the Caribbean

In many other parts of the Caribbean, like Trinidad [43], Jamaica [44] and Guyana [45], East Indian migrants became the primary food producers after their contract had finished. Their ethnobotanical legacy is substantial, as they introduced at least 75 botanical species to Jamaica [44], but in general little research has been done on the crop cultivars they brought from India. Given the recent decline in agricultural activities among Indo-Caribbeans and their rapid incorporation in mainstream society [43], it is important to trace their specific contribution to the agrodiversity in the Caribbean, and document the original varieties of the indentured laborers before they are lost forever. In contrast ro Suriname, (descendants of) Maroon communities in the Caribbean do not grow rice (anymore), so there is little chance of finding the ancient Indian landraces back on the farms of Afro-Caribbeans.

## Conclusions

Hindustani smallholders in Suriname have lost the traditional rice landraces that were brought along from India at the end of the nineteenth century by their ancestors. Some of these ancient landraces, however, may have been exchanged with Maroons in the past. Molecular studies are required to confirm whether ‘descendants’ of ancient Indian landraces still survive on Maroon rice fields, in particular those carrying names like Koelie aleisi, Mbotombolia, Patalika or Watralanti.

The Hindustani farmers we interviewed mostly grew modern rice cultivars that were developed by breeding institutes after 2000. Occasionally, they planted old cultivars from the 1950s for fodder, but samples from these seed stocks were heavily mixed with rice weeds and weedy rice. Still, fodder rice was sometimes sold for an inflated price as ‘ritual Hindu rice’ in the Netherlands.

Traditional cultivation practices, as described in the 1930s, have been abandoned. Bullocks have been replaced by tractors and the fierce competition by large-scale rice producers will probably soon lead to the disappearance of all small-scale rice farmers. Antique wooden farm equipment and processing tools were still encountered in people’s homes, thrift shops and the Lalla Rookh museum.

Most of our participants still retained pleasant memories of the manual planting, harvesting, and processing of rice, as well as the many gender-based practices and beliefs associated with the cultivation of the crop. In spite of their poverty and back-breaking work, people said they had been happy to be self-sufficient in food and enjoyed a strong sense of community and mutual support. Although rice still plays an important role in Hindu religion, rituals can now also be performed with shop-bought rice instead of home-grown padi. The oral history of former rice farmers, however, should be better preserved than it is today. Personal stories and traditional rice varieties (obtained from Maroon fields) could play a role in museum settings as living vehicles for memories of the descendants of Asian contract labourers in Suriname. The same applies for other traditional crop landraces grown by descendants of indentured Asians elsewhere in the Caribbean.

## Supplementary Information

**Additional file 1.** Historic rice varieties retrieved from literature and herbarium collections, mentioned as cultivated by East Indian contract laborers and small-scale farmers in Guyana and Suriname in the period 1863-present. All rice varieties belong to Oryza sativa L.

**Additional file 2.** Questionnaire.

**Additional file 3.** Rice varieties (modern and historic) mentioned during interviews, current and past growing location, agricultural and culinary characteristics, and presence in herbarium or germplasm collections.

**Additional file 4.** Vernacular rice terms mentioned during interviewsVernacular rice terms mentioned during interviews.

## Data Availability

Seed collections made during this research are available at Naturalis Biodiversity Center (Leiden, the Netherlands). Germplasm collected during this research is available at SNRI/ADRON in Nickerie, Suriname. The datasets generated and analyzed during the current study are available from the corresponding author on reasonable request.
