# Supplementary material for: The legacy of traditional rice cultivation by descendants of Indian contract laborers in Suriname
Source: J Ethnobiol Ethnomed. 2021 Oct 18;17:60. doi: 10.1186/s13002-021-00485-6 (PMC8524977; doi:10.1186/s13002-021-00485-6)
Supplement: Supplementary file 2 — Additional file 2. Questionnaire. [file 13002_2021_485_MOESM2_ESM.docx]

The legacy of traditional rice cultivation by descendants of Indian contract laborers in Suriname.

Supplementary Table 2. Questionnaire

| Subject | Question |
| --- | --- |
| Interview location | Country, City, Address |
| Personal data | Name, Gender, Age, Ethnicity |
|  | Current residence, Residence during rice cultivation |
|  | Who in your household cultivates/cultivated rice? |
| Location padi field | Geographic location |
|  | Estimation of rice field size |
|  | Type of rice cultivation wet/dry |
| Sowing material | How / where did you obtain the padi for sowing? |
|  | Was there ever rice exchanged with other ethnicities? |
|  | Was there rice brought from India? |
| Names of rice varieties | Free-listing of rice variety names |
|  | Recognition of rice names from preliminary database and sample book |
| Agronomic features | Morphological characteristics of rice plants and seeds |
|  | Growth time of varieties |
|  | Presence and explanation of red rice grains in harvest |
|  | Ecological requirements for growth |
|  | Affected by pests and diseases? |
|  | Positive / negative features of varieties |
| Uses | Fodder / home consumption / sale / rituals |
|  | Description of taste, use in specific recipes |
|  | Religious / spiritual beliefs associated with rice |
| Motivation | Why did you continue or abandon rice cultivation? |
|  | Own experience / personal story about rice cultivation? |
| Farming methods | Planting, growing, harvesting, processing (traditional vs. modern) |
|  | Other crops cultivated in the rice field |
|  | Use of fertilizer and pesticides |
|  | Gender-based division of labor in rice cultivation |
